# Screening of Pharmacologically Active Small Molecule Compounds Identifies Antifungal Agents Against *Candida* Biofilms

**DOI:** 10.3389/fmicb.2015.01453

**Published:** 2015-12-22

**Authors:** Takao Watamoto, Hiroshi Egusa, Takashi Sawase, Hirofumi Yatani

**Affiliations:** ^1^Department of Applied Prosthodontics, Graduate School of Biomedical Sciences, Nagasaki UniversityNagasaki, Japan; ^2^Division of Molecular and Regenerative Prosthodontics, Tohoku University Graduate School of DentistrySendai, Japan; ^3^Department of Fixed Prosthodontics, Osaka University Graduate School of DentistrySuita, Japan

**Keywords:** drug discovery, antifungal drug, biofilm, Small molecules, *Candida albicans*

## Abstract

*Candida* species have emerged as important and common opportunistic human pathogens, particularly in immunocompromised individuals. The current antifungal therapies either have toxic side effects or are insufficiently effect. The aim of this study is develop new small-molecule antifungal compounds by library screening methods using *Candida albicans*, and to evaluate their antifungal effects on *Candida* biofilms and cytotoxic effects on human cells. Wild-type *C. albicans* strain SC5314 was used in library screening. To identify antifungal compounds, we screened a small-molecule library of 1,280 pharmacologically active compounds (LOPAC^1280TM^) using an antifungal susceptibility test (AST). To investigate the antifungal effects of the hit compounds, ASTs were conducted using *Candida* strains in various growth modes, including biofilms. We tested the cytotoxicity of the hit compounds using human gingival fibroblast (hGF) cells to evaluate their clinical safety. Only 35 compounds were identified by screening, which inhibited the metabolic activity of *C. albicans* by >50%. Of these, 26 compounds had fungistatic effects and nine compounds had fungicidal effects on *C. albicans*. Five compounds, BAY11-7082, BAY11-7085, sanguinarine chloride hydrate, ellipticine and CV-3988, had strong fungicidal effects and could inhibit the metabolic activity of *Candida* biofilms. However, BAY11-7082, BAY11-7085, sanguinarine chloride hydrate and ellipticine were cytotoxic to hGF cells at low concentrations. CV-3988 showed no cytotoxicity at a fungicidal concentration. Four of the compounds identified, BAY11-7082, BAY11-7085, sanguinarine chloride hydrate and ellipticine, had toxic effects on *Candida* strains and hGF cells. In contrast, CV-3988 had fungicidal effects on *Candida* strains, but low cytotoxic effects on hGF cells. Therefore, this screening reveals agent, CV-3988 that was previously unknown to be antifungal agent, which could be a novel therapies for superficial mucosal candidiasis.

## Introduction

*Candida* species have emerged as important and common opportunistic human pathogens, particularly in immunocompromised individuals, such as patients with HIV/AIDS, patients with cancer undergoing chemotherapy, organ transplant recipients receiving immunosuppressive drugs and patients with advanced diabetes (Richardson, 2005; Aperis et al., 2006). *Candida* sp. are responsible for a spectrum of diseases, which range from local mucosal infections to life-threatening invasive systemic candidiasis (Wisplinghoff et al., 2004).

A key feature of the virulence of *Candida* sp. is their ability to adhere to surfaces, before developing into distinct surface-attached communities called biofilms. Biofilms may develop on biological and inert surfaces, such as intravascular catheters, stents, shunts, prostheses and implants (Raad, 1998; Ramage et al., 2006). *Candida* biofilms are intrinsically more resistant to commercially available antifungal agents than their planktonic counterparts (Hawser and Douglas, 1995; Chandra et al., 2001; LaFleur et al., 2006; Seneviratne et al., 2008). Thus, the biofilms that form on medical device can resist the host immune defenses and antifungal treatments, thereby causing chronic infections and failure of implanted medical devices (Ramage et al., 2005). The increasing number of immunocompromised patients and advances in medical technology has led to an increase in biofilm-related infectious diseases, where *Candida albicans* is the major fungal pathogen. Recently, the frequency of these candidiasis caused by the non *C. albicans* species of *Candida*, such as *C. glabrata, C. parapsilosis, C. dubliniensis*, and *C. tropicalis*, has increased due to the indiscriminate use of antifungal drugs (Cuellar-Cruz et al., 2012; Pfaller, 2012).

In addition, *C. glabrata, C. parapsilosis*, and *C. krusei* exhibit intrinsic resistance to most azole-based antifungal drugs (Lee et al., 2009a; Kothavade et al., 2010; Pfaller et al., 2011) and the emergence of acquired drug resistance to most commercial antifungals has been reported.(Sanglard and Odds, 2002; Pfaller et al., 2010). Despite the urgent requirement for efficient antifungal therapies of systemic infections, the available antifungal drugs, such as novel polyene formulations, new azoles and echinocandins, are few and expensive and have side effects (Rex et al., 2000; Francois et al., 2005; Cornely et al., 2007; Pasqualotto and Denning, 2008). Furthermore, common non-life-threatening superficial infections, such as recurrent vulvovaginal candidiasis, impose significant restrictions on patients and result in a reduced quality of life. Thus, it is necessary to develop new antifungal agents that are effective against *Candida* biofilms. These agents should overwhelm biofilm-related candidiasis and lead to more effective antifungal treatments.

In recent studies, library screening methods have been used to identify new antifungal agents, which have focused on growth retardation or killing the pathogens (LaFleur et al., 2011; Siles et al., 2013; Stylianou et al., 2014). This type of screening method can identify candidate antifungal agents from large numbers of small-molecule compounds. Small-molecule compounds have many advantages, such as simple synthesis, high chemical stability and low costs compared with organic compounds. Therefore, the aim of the present study was to develop new small-molecule antifungal compounds by library screening methods using *C. albicans*. Moreover, we evaluated the antifungal effects of the small molecules detected by the library screening method using *Candida* biofilms as well as their cytotoxic effects on human cells.

## Materials and Methods

### Drugs and Fungal Strains

The *in vitro* susceptibility of well-characterized wild-type *C. albicans* strain SC5314, which was provided by Prof. N.A.R. Gow (University of Aberdeen, Aberdeen, UK) was tested against 1280 compounds from the Library of Pharmacologically Active Compounds (LOPAC^1280TM^, Sigma–Aldrich, USA). The screen was performed with *C. albicans* SC5314, and hits were further confirmed with the type strains *C. dubliniensis* MYA 577, *C. glabrata* ATCC 2001, *C. kusei* ATCC 6258, *C. palapsilosis* ATCC 22019, and *C. tropicalis* ATCC13803.

### High-Throughput Screening (HTS) with Antifungal Susceptibility Tests (ASTs)

**FIGURE 1:**
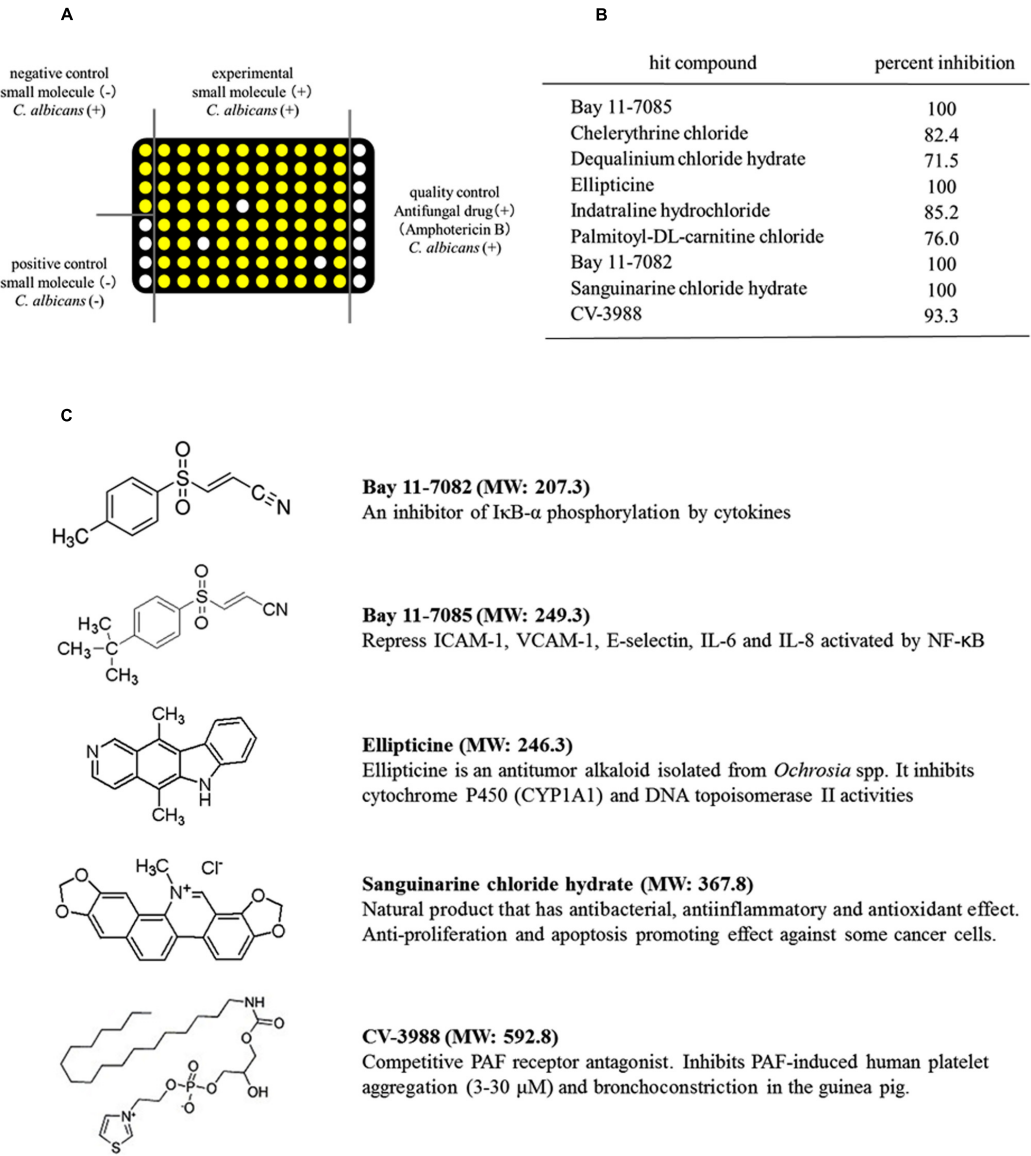
**Identification of small-molecule compounds that inhibited the metabolic activity of *Candida albicans* using high-throughput screening (HTS). (A)** Schematic showing the HTS procedure. White circles: low ATP level and no metabolic activity in *C. albicans.* Yellow circles: high ATP level and high metabolic activity in *C. albicans.* **(B)** Compounds that inhibited the metabolic activity of *C. albicans*. **(C)** Structures of the five compounds that inhibited the metabolic activity of *C. albicans* by >90%. MW, molecular weight.

High-Throughput Screening was conducted using ASTs, according to the standard Clinical and Laboratory Standard Institute (CLSI) method (Watamoto et al., 2009). Inocula from 24-h yeast cultures on Sabouraud’s dextrose agar (SDA) (Gibco, UK) were adjusted to a turbidity equivalent to a 0.5 McFarland standard at 520 nm using a spectrophotometer. The suspension was diluted further with RPMI 1640 medium (Gibco, UK) to yield an inoculum concentration of 0.5 × 10^3^ to 2.5 × 10^3^ cells/mL. *C. albicans* was incubated with small-molecule compounds (10 μM) from LOPAC^1280TM^, which total volume was 150 μL, in 96-well plates at 37°C for 24 h to evaluate the antifungal effects. After incubation, the viability of the fungal cells was determined using the CellTiter-Glo luminescent cell viability kit (Promega, USA). The CellTiter-Glo reagent (150 μL) was added to the medium and incubated for 15 min at room temperature with shaking at 900 rpm. The luminescent signals were detected using a luminometer (GloMax Discover System, Promega, USA). The resulting signal intensity corresponds to ATP amounts and thus to the number of viable microbial cells upon drug exposure (Stylianou et al., 2014). In all 96-well plates, 100 and 0% growth controls were included as microbes plus dimethyl sulfoxide (0.1%) and microbes plus amphotericin B (100 μM), respectively. All assays were performed at least as two biological replicates in triplicate. The ATP level of *C. albicans* cells, which corresponded to the cell metabolic activity and viability, was calculated for each compound using the following equation (**Figure 1A**).

$$\mathrm{Percentage}\mathrm{inhibition}=100\;\times \;1-\left\{\frac{\left(experimental-positive\mathrm{control}\mathrm{average}\right)}{\left(\mathrm{negative}\mathrm{control}average-positive\mathrm{control}\mathrm{average}\right)}\right\}$$

Wells were scored as hits if the percentage inhibition was >50%. Hit compounds were evaluated further to assess their antifungal effects.

### ASTs of Hit Compound in Various Growth Modes Against *Candida* Strains

To investigate the antifungal effects of the hit compounds, ASTs were conducted using broth microdilution assays with high cell densities of the planktonic mode, adhesion phase and biofilm mode against *Candida* strains (*C. albicans, C. dubliniensis, C. glabrata, C. kusei, C. palapsilosis*, and *C. tropicalis*). First, high density cell (1 × 10^7^ cells/mL) suspensions were added to the RPMI medium containing each hit compound (10–1000 μM) in 96-well plates and incubated at 37°C for 24 h. Next, the 50% minimum inhibitory concentrations (MICs) of high-density *Candida* planktonic cultures were determined using the CellTiter-Glo luminescent cell viability kit, as described above. The antifungal effects of the hit compounds were also evaluated in the adhesion phase and the biofilm mode, in the same manner as the planktonic mode. *Candida* biofilms were produced as described previously (Jin et al., 2004). In brief, *Candida* cells were grown on SDA at 37°C for 18 h. A loopful of the yeast culture was then inoculated into yeast nitrogen base (YNB) (Difco, USA) medium supplemented with 50 mM glucose. After overnight broth culture in a rotary shaker at 75 rpm, the cells were washed twice with 20 mL of PBS (pH 7.2, 0.1 M). The yeast cells were re-suspended in YNB supplemented with 100 mM glucose and adjusted to an optical density of 0.38 (1 × 10^7^ cells/mL) at 520 nm. This standardized cell suspension was used immediately to form biofilms in the wells of 96-well polystyrene culture plates (Iwaki, Tokyo, Japan). First, the cells were incubated for 90 min at 37°C in a shaker at 75 rpm to allow yeast adherence to the well surface (adhesion phase), before the medium was aspirated and each well was washed once with PBS to remove non-adherent cells. YNB containing 100 mM glucose was then pipetted into each well and the plate was incubated at 37°C in a shaker at 75 rpm for 24 h. Non-adherent cells were removed by pipetting and the biofilms were washed twice with PBS. Following this biofilm growth phase, microscopic examination of the cultures was performed to exclude contamination. These ASTs were repeated on three different occasions.

### Cytotoxicity

Primary human gingival fibroblast (hGF) cultures were established from discarded healthy gingival tissues after surgery with the informed consent of the donors (Nikawa et al., 2006). In brief, the gingival tissue specimens were treated overnight with 0.025% trypsin and 0.02% EDTA at 4°C. After trypsin neutralization, the lamina propria mucosae were separated from the epithelial layer and minced into pieces in a plastic tissue culture dish, and then maintained in Dulbecco’s modified Eagle medium (Nacalai Tesque, Kyoto, Japan) supplemented with 10% FBS, 100 U/mL penicillin, 100 mg/mL streptomycin and 250 ng/mL amphotericin B (Nacalai Tesque, Kyoto, Japan). After the fibroblasts had migrated out of the tissue, the tissues were removed and the cells were cultured until they reached confluence. The cells were then seeded onto 96-well tissue culture plates (500 cells per well) and the culture medium was exchanged with fresh growth medium containing the hit compounds (0.98–1000 μM). The cells were cultured continuously and the culture medium containing the hit compounds was renewed every other day. The number of cells was evaluated using the WST-1 cell counting assay (Dojindo Laboratories, Kumamoto, Japan), as described previously (Hamada et al., 2007). The highest concentration of each compound that caused greater than 50% reduction in the number of cell compare to that of compound free control cell was reported as the cytotoxic concentration. All the experiments were performed using three samples for each condition in triplicate.

## Results

### High-Throughput Screening (HTS) Results

We screened 1280 compounds using antifungal susceptibility tests (ASTs) in 96-well plates to identify antifungal agents. Only 35 compounds were identified, which inhibited the metabolic activity of *C. albicans* by >50%. Thus, the overall hit rate for HTS was approximately 3.9%. Among the hit compounds, 26 compounds had fungistatic effects and nine compounds had fungicidal effects on *C. albicans* (**Figure 1B**). Five compounds, BAY11-7082, BAY11-7085, sanguinarine chloride hydrate, ellipticine and CV-3988, had strong fungicidal effects and inhibited the metabolic activity of *C. albicans* by >90% (**Figure 1B**). The structures of these five compounds are shown in **Figure 1C**. The antifungal effects of these five compounds were evaluated using *Candida* strains (*C. albicans, C. dubliniensis, C. glabrata, C. kusei, C. palapsilosis*, and *C. tropicalis*) in high density planktonic, adhesion and biofilm modes.

### ASTs of Hit Compounds Using *Candida* Strains in Various Growth Modes

The HTS results showed that *C. albicans* was susceptible to all the hit compounds when a low inoculum size (1 × 10^3^ cells/mL) was used, according to the CLSI methodology (MIC < 1 μM). When the cell density increased to 1 × 10^7^ cells/mL, *Candida* strains were slightly resistant to four of the compounds, but not sanguinarine chloride hydrate. However, all five compounds inhibited the metabolic activity of *Candida* strains at <31.3 μM and they had fungicidal effects on the high cell density planktonic mode (**Table 1**). As a control, amphotericin B inhibited the metabolic activity of *C. albicans* at <3.9 μM.

**Table 1:** Minimum inhibitory concentrations (MICs) of five candidate compounds against planktonic mode of *Candida* strains.

	*C. albicans*	*C. glabrata*	*C. dubliniensis*	*C. tropicalis*	*C. kusei*	*C. palapsilosis*
Bay11-7082	7.8	7.8	3.9	7.8	3.9	3.9
Bay11-7085	3.9	3.9	3.9	3.9	3.9	3.9
Sanguinarine	<1	<1	<1	<1	<1	<1
Ellipticine	7.8	7.8	3.9	15.6	7.8	7.8
CV-3988	7.8	7.8	31.3	31.3	15.6	15.6
AMB	3.9					

						(μM)

*Sanguinarine, sanguinarine chloride hydrate; AMB, amphotericin B; MICs, minimal concentration of compound resulting in >50% growth inhibition. MICs were determined by ATP measurement after 24 h of incubation. The data were analyzed and evaluated from 3 biological replicate in triplicate (*n* = 3).*

The drug susceptibility of adhesion phase *Candida* strains to the five compounds was higher than that of the high density planktonic cultures (**Table 2**). In particular, sanguinarine chloride hydrate was effective against adhesion phase and it could inhibit the metabolic activity at <15.6 μM. Bay 11-7082 and Bay 11-7085 were also effective against the adhesion phase and could inhibit the metabolic activity at <31.3 μM. As a control, amphotericin B inhibited the metabolic activity of *C. albicans* adhesion phase at 15.6 μM.

**Table 2:** Minimum inhibitory concentrations of five candidate compounds against adhesion phase of *Candida* strains.

	*C. albicans*	*C. glabrata*	*C. dubliniensis*	*C. tropicalis*	*C. kusei*	*C. palapsilosis*
Bay11-7082	31.3	31.3	15.6	31.3	15.6	15.6
Bay11-7085	31.3	31.3	15.6	31.3	15.6	7.8
Sanguinarine	15.6	15.6	15.6	7.8	7.8	7.8
Ellipticine	62.5	62.5	125	250	250	125
CV-3988	62.5	62.5	125	62.5	125	125
AMB	15.6					

						(μM)

*Sanguinarine, sanguinarine chloride hydrate; AMB, amphotericin B; MICs, minimal concentration of compound resulting in >50% growth inhibition. MICs were determined by ATP measurement after 24 h of incubation. The data were analyzed and evaluated from 3 biological replicate in triplicate (*n* = 3).*

Most *Candida* biofilms were more resistant to the five compounds than other growth mode. Especially, *C. tropicalis* biofilm was most resistant to the five compounds in all growth modes (**Table 3**). Bay 11-7082, Bay 11-7085, Ellipticine and CV-3988 could inhibit the metabolic activity of *Candida* biofilms at <62.5, 62.5, 500, and 125 μM, respectively. Sanguinarine chloride hydrate was the most effective antifungal agent in this study and it could inhibit the metabolic activity of *Candida* strains at <31.3 μM. As a control, amphotericin B inhibited the metabolic activity of *C. albicans* biofilm at 62.5 μM.

**Table 3:** Minimum inhibitory concentrations of five candidate compounds against biofilm mode of *Candida* strains.

	*C. albicans*	*C. glabrata*	*C. dubliniensis*	*C. tropicalis*	*C. kusei*	*C. palapsilosis*
Bay11-7082	31.3	62.5	31.3	62.5	62.5	15.6
Bay11-7085	31.3	62.5	62.5	62.5	62.5	15.6
Sanguinarine	15.6	15.6	15.6	31.3	31.3	7.8
Ellipticine	125	62.5	500	250	250	250
CV-3988	125	125	125	125	125	125
AMB	62.5					

						(μM)

*Sanguinarine, sanguinarine chloride hydrate; AMB, amphotericin B; MICs, minimal concentration of compound resulting in >50% growth inhibition. MICs were determined by ATP measurement after 24 h of incubation. The data were analyzed and evaluated from 3 biological replicate in triplicate (*n* = 3).*

### Cytotoxicity

In addition to pharmacologically active compounds, small-molecule libraries often contain toxic molecules that do not make good drug candidates. To evaluate the safety for clinical use, we tested the cytotoxic effects of the hit compounds using human cell cultures. We used hGF cells because of their ubiquitous nature and their widespread use in cytotoxicity testing (Egusa et al., 2009; LaFleur et al., 2011). The hGF cells were grown in 96-well plates and exposed to increasing doses (two-fold increments) of each hit compound for 4 days. The hGF metabolic activity was measured every other day and used as an indicator of cell viability. After 4 days, Bay 11-7082, Bay 11-7085, ellipticine, sanguinarine chloride hydrate and CV-3988 inhibited cell proliferation no more than 50%, namely, did not kill cells at less than 7.81, 7.81, 1.95, 0.73, and 250 μM, respectively (**Table 4**).

**Table 4:** Cytotoxic concentrations of five candidate compounds on human gingival fibroblasts.

	Cytotoxic concentration
Bay11-7082	7.81
Bay11-7085	7.81
Ellipticine	1.95
Sanguinarine chloride hydrate	0.73
CV-3988	250

	(μM)

*Cytotoxic concentration, maximal concentration of compound resulting in >50% the number of cell reduction compares to compound free control. The data were analyzed and evaluated from 3 biological replicate in triplicate (*n* = 3).*

## Discussion

*Candida* species are the main fungal pathogen that causes infections in humans, ranging from superficial mucosal infection to systemic mycoses (Navarro-Garcia et al., 2001). *Candida* infections are intractable and recurrent diseases, which have increased due to the rise in the number of immunocompromised host populations (Beck-Sague and Jarvis, 1993; Wisplinghoff et al., 2004). Drug-resistant *Candida* strains have also increased dramatically because of the increased use of antifungal agents. Thus, the development of novel antifungal drugs and treatment strategies are essential for combating *Candida* infections. High-throughput screening (HTS) is an effective method for identifying candidate novel antifungal drugs. It is important to apply adequate screening methods to small-molecule compound libraries because appropriate selection procedures are the key to successful screening. In this study, LOPAC^1280TM^ was used as the small-molecule library, which contained pharmacologically active compounds and all the compounds were commercially available. Thus, the main effects of these small molecules on human cells are already known and described in database of manufacture. Therefore, it may be easier to apply these compounds in clinical practice with fewer unexpected drug side effects.

In general, polyenes, azoles, allylamines, morpholines, antimetabolites, and echinocandins are the six major antifungal drug categories to manage fungal infections (Khan and Jain, 2000; Ruhnke et al., 2008). Most of these antifungal drugs have fungistatic or fungicidal effects on exponentially growing planktonic cells, but *Candida* cells are resistant to these drugs after biofilm formation (Watamoto et al., 2009). Interestingly, we found that five small-molecule compounds (BAY11-7082, BAY11-7085, sanguinarine chloride hydrate, ellipticine and CV-3988) were antifungal drug candidates with inhibitory effects on various *Candida* biofilms at concentrations below 500 μM.

BAY11-7082 and BAY 11-7085 is known to be an inhibitor of nuclear factor κB (NF-κB) activation by the blockade of inhibitor κB (IκB) phosphorylation, which is a trigger of apoptosis (Pierce et al., 1997; Guzman and Jordan, 2005; Chopra et al., 2008; Lee et al., 2009b; Zanotto-Filho et al., 2010). Bay 11-7082 triggers cell membrane scrambling and cell shrinkage (Lang et al., 2008). BAY 11-7085 has been shown to activate c-jun N-terminal kinase and p38 mitogen-activated protein kinase (MAPK) (Pierce et al., 1997). BAY 11-7085 inhibits cell proliferation by inducing apoptosis and G0/G1 arrest of the cell cycle in human cells (Bockelmann et al., 2005). These actions have anti-inflammatory, anticancer and slight hemolytic effects (Ghashghaeinia et al., 2011).

Sanguinarine chloride hydrate is a phytoalexin and has been reported to suppress activation of the transcription factor NF-κB (Chaturvedi et al., 1997) and to modulate the functions of various enzymes, such as MAPK phosphatase-1 (Vogt et al., 2005), protein kinase C (Gopalakrishna et al., 1995) and phosphoinositide-dependent protein kinase 1 (Vrba et al., 2008). These actions of Sanguinarine have antimicrobial, antioxidant, anti-inflammatory, hemolytic and cytotoxic effects (Lenfeld et al., 1981; Godowski, 1989; Malikova et al., 2006; Babu et al., 2008; Matkar et al., 2008; Jang et al., 2009).

Ellipticine, an alkaloid isolated from Apocyanaceae plants, has been reported to mediate primarily DNA damage such as DNA intercalation (Auclair, 1987), inhibition of topoisomerase II (Auclair, 1987; Stiborova et al., 2006), inhibition of casein kinase 2 (Prudent et al., 2010) and the formation of covalent DNA adducts by cytochrome P450s and peroxidases (Stiborova et al., 2011). These actions of Ellipticine has anti-tumor, cytotoxic, hemolytic and mutagenic activities (Lee, 1976; Rouesse et al., 1985). Therefore, the known cell proliferation inhibitory effects of these four small-molecules agree with the findings of the present study. Furthermore, the antifungal and cytotoxic effects of these small molecules on *Candida* strains may involve the same mechanism because *Candida* strains are eukaryotes and possesses the same targets. Thus, these small molecules are toxic to human cells and *Candida* strains, and inappropriate for clinical use corroborated by the relatively low cytotoxic concentration on hGF.

On the other hand, platelet-activating factor (PAF), which is released almost immediately in response to inflammatory stimuli (Im et al., 1997) by various inflammatory cells, is a potent lipid messenger involved in cellular activation, fertilization, intracellular signaling, apoptosis and diverse inflammatory reactions (Braquet et al., 1987; Shukla, 1992; Buttke and Sandstrom, 1995; Fukuda and Breuel, 1996). CV-3988 (Terashita et al., 1983; Terashita et al., 1987) is a structural analog of PAF, which has been shown to specifically inhibit the *in vitro* and *in vivo* activities of PAF (Sultana et al., 1999) by competitive binding with the PAF receptor (PAF-R) (Terashita et al., 1983; Summers and Albert, 1995; Negro Alvarez et al., 1997). Therefore, CV-3988 is an antagonist of PAF-R, which inhibits the functions of leukocytes, including platelet aggregation, inflammation and anaphylaxis. We showed for the first time that CV-3988 had a fungicidal effect on various *Candida* biofilms and low cytotoxity effect on hGF cells. In past study, CV-3988 had slight hemolytic effect and can safely be administered to human (Arnout et al., 1988). These results demonstrate that CV-3988 has a novel and specific fungicidal effect on *Candida* strains and may become initial drug choice for the treatment of candidiasis. Furthermore, *Candida* sp. are common microbes in the oral cavity and vagina and causes mucotitis in immunocompromised and healthy hosts. Mouthwashes and ointments containing antifungal agents are primary treatment for oral and vaginal candidiasis. Therefore, CV-3988 may be suitable for use on oral mucosal surfaces to combat *Candida* biofilm infections such as thrush and denture-related stomatitis. Although CV-3988 may facilitate novel treatment strategies to combat *Candida* infections, further studies about fungicidal mechanism and pharmacokinetics are required before it can be applied in clinical practice.

## Conclusion

We identified five small-molecule compounds (BAY11-7082, BAY11-7085, sanguinarine chloride hydrate, ellipticine and CV-3988) as novel antifungal drug candidates using HTS methods. BAY11-7082, BAY11-7085, sanguinarine chloride hydrate and ellipticine were toxic to *Candida* strains as well as hGF cells. In contrast, CV-3988 had fungicidal effects on *Candida* strains, but low cytotoxic effects on hGF cells. Therefore, in future, mouthwashes and ointments containing CV-3988 may be used as a novel treatment for superficial mucosal candidiasis.

## Conflict of Interest Statement

The authors declare that the research was conducted in the absence of any commercial or financial relationships that could be construed as a potential conflict of interest.
